# Integrating outdoor environments into health service delivery at residential care facilities for older adults

**DOI:** 10.1186/s12913-026-15852-w

**Published:** 2026-10-14

**Authors:** Madeleine Liljegren, Anna Bengtsson, Göran Lindahl, Helle Wijk

**Affiliations:** 1 Institute of Health and Care Sciences, University of Gothenburg, Arvid Wallgrens Backe, House 1, Box 457, Gothenburg, 405 30 Sweden; 2 Division of Construction Management and Engineering, Department of Architecture and Civil Engineering, Chalmers University of Technology, Sven Hultins Street 6, Gothenburg, 412 96 Sweden; 3 Department of People and Society, The Swedish University of Agricultural Sciences, Slottsvägen 5, Alnarp, 234 56 Sweden

**Keywords:** Care worker, Everyday activities, Older adult, Outdoor environment, Person-centred care and rehabilitation, Residential care facility, Healthcare delivery

## Abstract

**Background:**

Outdoor environments may support everyday activities, person-centred care and rehabilitation, and health and well-being among older adults in residential care facilities (RCFs). However, how they can be integrated into routine health service delivery remains underexplored. This study aimed to explore how outdoor environments can support everyday activities and person-centred care and rehabilitation in RCFs, and to identify perceived benefits and barriers to their use in routine health service delivery.

**Methods:**

A qualitative study was conducted using individual walking interviews with older adults and focus group walking interviews with care workers at three Swedish RCFs. Data were organised according to the model of four zones in contact with the outdoors: indoor environments with windows (Zone 1), transitional outdoor environments (Zone 2), gardens (Zone 3), and the surroundings (Zone 4). Everyday activities, care and rehabilitation were identified across the zones, together with perceived benefits and barriers. Practices were distinguished as either currently occurring or potential uses.

**Results:**

All four zones offered opportunities for everyday activities, care and rehabilitation, including social interaction, recreation, physical activity, contact with nature, health conversations, and assessments. Perceived benefits included stimulation, enjoyment, social interaction, sensory experiences, and contact with nature. However, use was limited by physical, seasonal, organisational, informational, and social barriers. The findings indicate a gap between the opportunities outdoor environments offer and their integration into everyday routines.

**Conclusion:**

Outdoor environments are a valuable but underutilized resource in RCFs. Their integration into routine health service delivery requires access to accessible environments, supportive organisational routines, and care workers with sufficient time, knowledge, and support. The identified practices across the four zones may provide practical inspiration for using outdoor environments in everyday activities and person-centred care and rehabilitation.

## Introduction

Despite the profound health and well-being benefits of outdoor stays, older adults and care workers often spend much of their time indoors. Contact with nature, both visual from inside the building and being outdoors, has well-documented health and well-being benefits among persons in need of health care. Integrating outdoor environments into health service delivery) can improve recovery, reduce medication use [1], foster emotional connectedness [2], and improve quality of life while lowering stress [3, 4]. Access to natural daylight further promotes health and well-being [5].

Outdoor stays and garden-based activities also contribute to positive health outcomes. They can reduce agitation, depression, stress [6], and sedentary behaviour [7, 8]. For older adults with cognitive decline, safe and accessible outdoor environments support independent movement [9] and can evoke fascination and psychological restoration [10, 11]. The most vulnerable older adults often benefit the most from these environments [12, 13]. Recent research further highlights that activity-friendly outdoor environments can compensate for functional impairments in older adults with disabilities by supporting remaining physical and psychological capacities, thereby facilitating outdoor activities and contributing to improved overall functioning [14]. However, environmental factors such as cold, heat, darkness, slippery surfaces, and pollen exposure can also hinder outdoor use [15]. In addition, international expert recommendations for older adults at residential care facilities (RCFs) emphasize the importance of reducing sedentary behaviour and providing evidence-based physical activity and exercise opportunities that account for older adults’ functional and cognitive abilities, while also fostering motivation and pleasure [16].

Employees may also benefit from contact with nature. Among office workers, access to views of natural environments from the workplace has been associated with greater work ability and job satisfaction [17]. Similarly, exposure to natural light and outdoor views has been associated with fewer medication errors among healthcare workers [18]. Access to natural light and outdoor views is also recognized as important for health and is recommended in international standards [19] and Swedish government authority documents [20], while insufficient exposure may adversely affect sleep and mood. Integrating outdoor activities into daily work routines can further promote better communication between care workers and older adults with cognitive decline [21] and reduce work-related stress during lunch breaks [22]. Furthermore, working outdoors can support recovery and well-being among employees [23]. Given these health benefits, it is particularly important to consider how outdoor environments can be integrated into health service delivery in RCFs to support older adults’ health and well-being, as well as care workers’ work practices.

### Living and working in RCFs

In some countries, such as Sweden, public authorities are commonly responsible for ensuring that older adults have access to housing, support, care, and rehabilitation. Although governance structures vary, RCFs constitute a central component of long-term care systems internationally. The physical environment in RCFs forms a key aspect for both facilitating older adults’ engagement in everyday activities and outdoor environments and delivering care and rehabilitation, combining shared spaces for social interaction with private, adapted apartments [24]. Care and rehabilitation are delivered by social care workers, such as assistant nurses (give 24-hour personal support) and activity leaders (support with social and physical activities), and healthcare workers, including nurses, occupational therapists, and physiotherapists. Services typically encompass personal care, medical treatment, rehabilitation, prescription of mobility aids, and support in daily activities [25]. The terminology and specific responsibilities of these roles may vary across countries. Workforce challenges in long-term care are widely documented, including high sickness absence, recruitment difficulties, and retention problems across national contexts [26, 27]. These challenges are linked to low job quality, including low pay, high workloads, stressful and physically demanding working conditions, limited career opportunities, and insufficient training relative to the increasingly complex tasks performed by LTC workers [28]. Consequently, strengthening work environments and developing care and rehabilitation practices, such as integrating outdoor activities, have been identified as important strategies to improve recruitment, retention, and job satisfaction [29]. Although situated within a specific national context, this study addresses structural conditions and workforce challenges that characterize RCFs internationally [28].

### Theoretical perspectives

This study draws on two complementary theoretical perspectives: person-centredness and the principal model of four zones in contact with the outdoors. Person-centredness provides the overarching perspective for understanding everyday activities, care, and rehabilitation, while the zone model provides a framework for understanding how different types of environmental access may support these practices. Within this study, outdoor everyday activities and person-centred care and rehabilitation are understood as part of health services, as they may constitute organised efforts to support health and well-being in RCFs.

Person-centredness is an ethical approach that recognises the person as an active participant in decisions concerning their health and everyday life. It is grounded in listening to and understanding the person, establishing a relationship, shared decision-making, and reaching a shared understanding regarding care [30, 31]. Person-centred care and person-centred rehabilitation [32] apply these principles to care and rehabilitation, respectively, with attention to the person’s needs, preferences, values, goals, resources, and participation. The practical implementation of person-centredness can be understood through four interrelated areas: prerequisites, referring to care workers’ knowledge, skills, and values; practice environment, encompassing physical, organisational, and psychosocial conditions; person-centred processes, referring to practices that respond to the person’s values, preferences, and needs; and outcomes, including well-being, satisfaction, and involvement. In this study, these areas provide an analytical lens for examining conditions and practices that may support person-centred everyday activities, care, and rehabilitation. Everyday activities refer to activities that form part of older adults’ daily lives, including routines, meals, social interaction, leisure, and meaningful engagement. Care refers to nursing and health-related practices addressing health, safety, and well-being, whereas rehabilitation refers to goal-directed practices aimed at maintaining or improving functional capacity and activity. These domains are analytically distinct but may overlap in practice; for example, outdoor walking may constitute both an everyday activity and a rehabilitative practice.

**Fig. 1:**
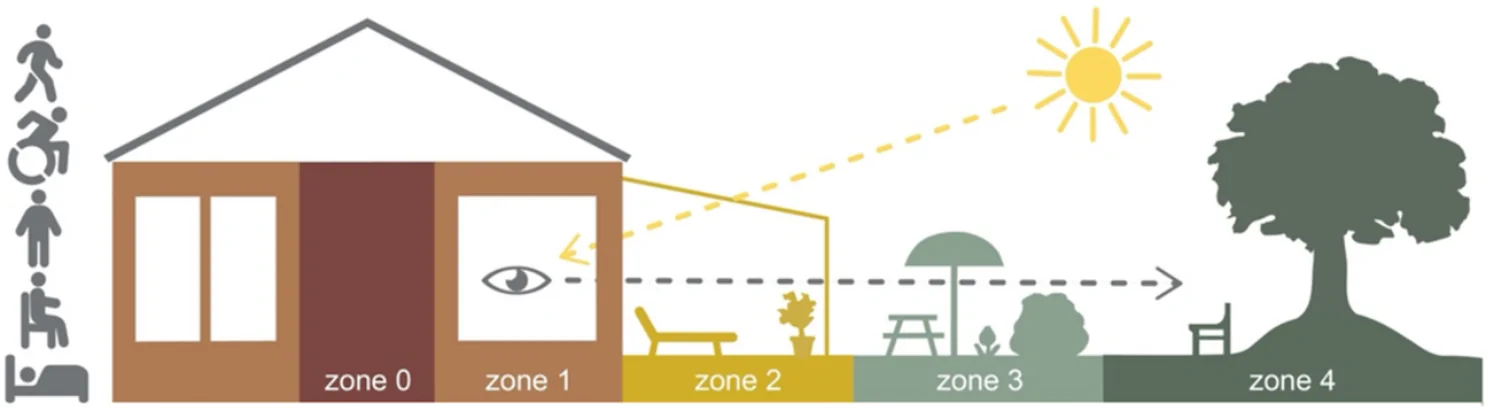
The zone model. Illustration A. Bengtsson [33]

The Principal model of four zones of contact with the outdoors [the zone model) conceptualizes environmental access: Zone 1 comprises indoor spaces with visual contact with the outdoors through windows (preferable natural elements); Zone 2 includes transitional areas such as balconies and patios; Zone 3 refers to gardens; and Zone 4 the surrounding environment [33]. In this study, Zone 1 referred to indoor environments, including older adults’ apartments and shared living rooms, activity rooms, and dining rooms, as well as care workers’ offices, staff rooms, rest rooms, and conference rooms. Zone 2 included transitional spaces such as entrances, balconies, patios, and conservatories (a fully glazed room in close proximity to the garden). Zone 3 referred to gardens, while Zone 4 comprised the surrounding outdoor environment, such as parks and public squares. The zones represent different types of potential contact with the outdoors and are conceptual rather than defined by specific visual relationships between the zones or the building. Each zone is associated with specific health-promoting qualities for both older adults and care workers, making access to all zones a relevant structural consideration [34]. The model considers older adults’ functional capacity and cognitive status, as these aspects may influence their ability to access and use different zones. The model also concerns care workers, as they support older adults in accessing and using the zones as part of everyday activities, and person-centred care and rehabilitation. Thus, the zones are relevant to both older adults’ opportunities for outdoor contact and care workers’ ability to facilitate such contact [35] (Fig. 1). In this study, the zone model is used to structure the description and analysis of everyday activities, care, and rehabilitation in relation to different types of environmental access. The model thereby complements the person-centred approach by providing an environmental structure through which person-centred practices can be examined.

Previous research has combined these perspectives to demonstrate how physical design shapes conditions for everyday outdoor activities and person-centred care and rehabilitation outdoors [35]. Despite documented health benefits of outdoor environments, older adults in RCFs spend most of their time indoors [36, 37]. Knowledge remains limited regarding how the different zones can be systematically used to support older adults’ everyday activities and person-centred care and rehabilitation practices.

## Methods and materials

The aim of this study is to explore how outdoor environments can support everyday activities and person-centred care and rehabilitation in RCFs, and to identify perceived benefits and barriers to their use in routine health service delivery.

This interdisciplinary study integrated knowledge from caring science, architecture, and landscape architecture. A qualitative descriptive design based on semi-structured interviews [38] was employed, using walking interviews [39], conducted individually with older adults and as focus group interviews with the care workers [40]. This methodological combination enabled participants to collaboratively explore aspects of care and rehabilitation in relation to the environment. The study’s findings are reported in accordance with the consolidated criteria for reporting qualitative research (COREQ) [41]. The study is a secondary analysis of the original sample and empirical data reported in two previous publications [42, 43], which addressed different research questions and analytical perspectives. The present study focuses on outdoor environments in relation to everyday activities, care, and rehabilitation.

### Settings

**Fig. 2:**
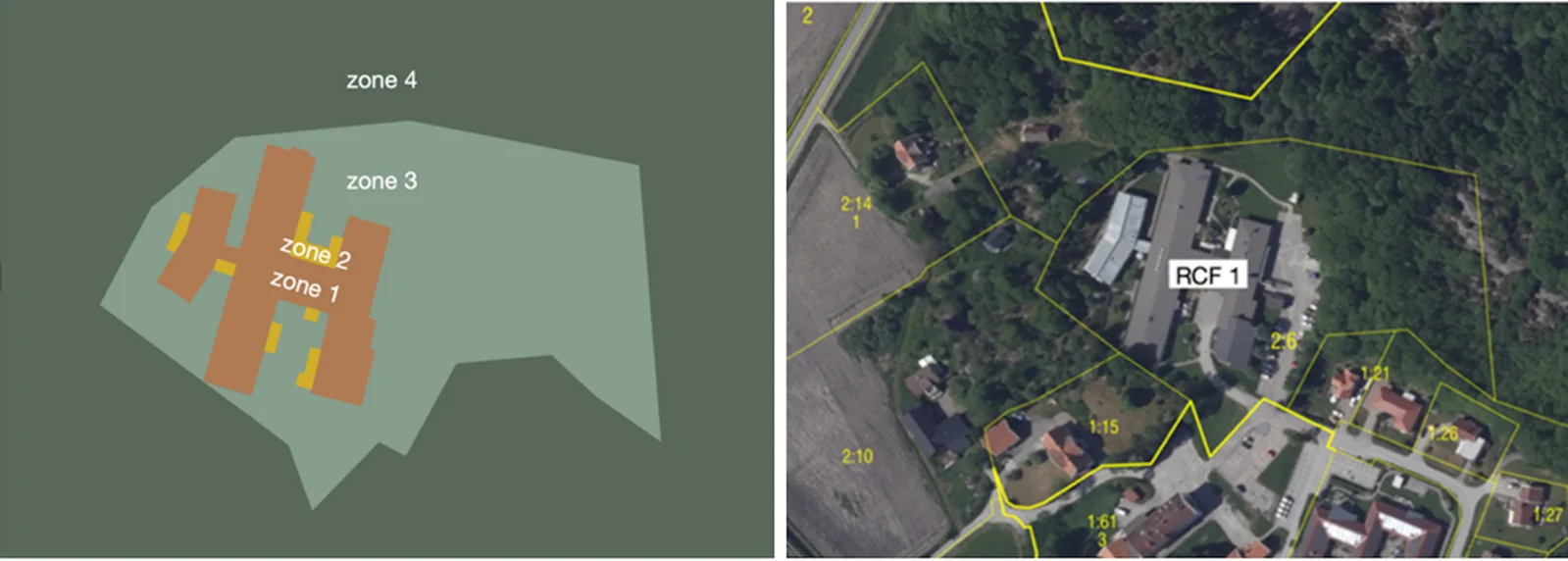
RCF 1 illustrated according to the zone model (left) and aerial view with marked plot boundaries (right). Illustrations by M. Liljegren. reproduced from Liljegren (2025) with permission

**Fig. 3:**
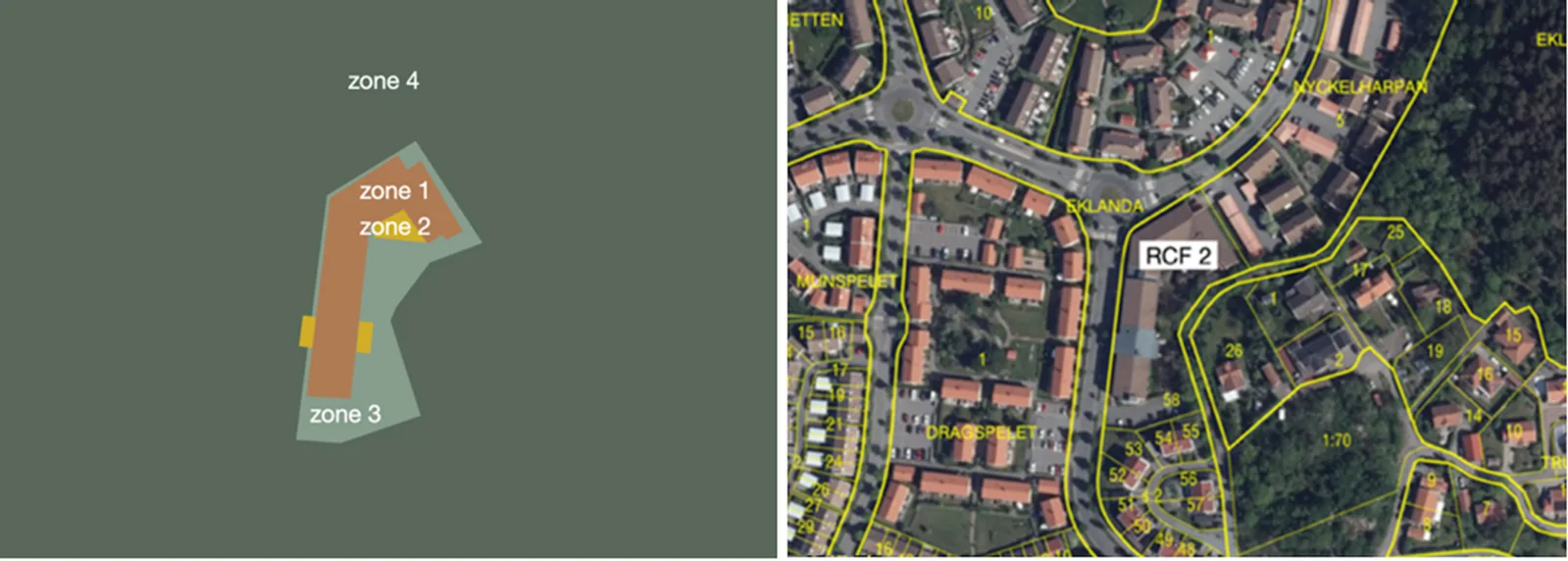
RCF 2 illustrated according to the zone model (left) and aerial view with marked plot boundaries (right). Illustrations by M. Liljegren. reproduced from Liljegren (2025) with permission

**Fig. 4:**
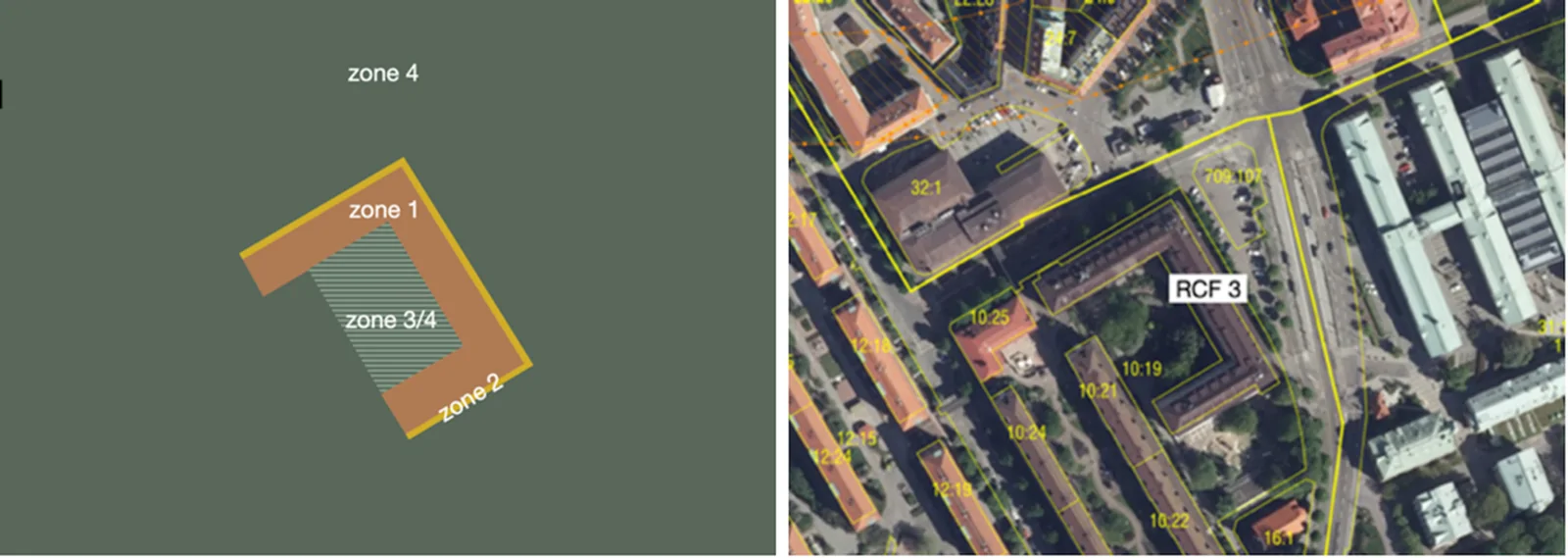
RCF 3 illustrated according to the zone model (left) and aerial view with marked plot boundaries (right). Illustrations by M. Liljegren. reproduced from Liljegren (2025) with permission

To ensure variation in access to the four zones, three Swedish RCFs were purposefully selected from municipalities of varying sizes and settings, ranging from a small village to a large city. These RCFs also differed in building height (one, three, and nine floors) and in the number of older adults and care workers. RCF 1, located on the outskirts of a small village, was a single-storey facility with a garden extending around the building. RCF 2, situated in a community close to a large city, comprised three storeys and provided access to a backyard garden. RCF 3, located in a large city, was a nine-storey facility with access to a park-like garden shared with other organisations. Figures 2, 3 and 4 illustrate the three RCFs according to the zone model structure and aerial views with plot boundaries.

### Participants

The participants consisted of older adults and care workers from the three RCFs. Purposive sampling of older adults was conducted to ensure diversity in body position/functional capacity and health status. Four older adults were selected for invitation from each RCF, resulting in a total of twelve resident participants. Mobility aids included walkers and both manual and electric wheelchairs. Severe cognitive decline was an exclusion criterion. Participant characteristics of older adults are detailed in Table 1.

**Table 1:** Description of older adults

Age	Number of older adults	Body positions /functional capacity	Number of older adults	Years living in RCFs	Number of older adults	Residential floors	Number of older adults
71–80 years old	2	In motion/ standing independently	2	Less than one year	2	Floor 1	4
81–90 years old	4	In motion/ standing, used walker independently	3	One year	3	Floor 2	2
91 years old or older	6	In motion/ sitting, used wheelchair independently	3	Two years	2	Floor 3	3
		In motion/ sitting, used wheelchair, dependent on personal support	4	More than two years	5	Floor 4	1
						Floor 5	1
						Floor 6	1

The care workers represented a range of professional roles. To capture a broad spectrum of experiences, purposive sampling was conducted based on profession (social care workers and healthcare workers), years of experience in RCFs, age, and gender. Eleven care workers participated in three focus group walking interviews (see Table 2).

**Table 2:** Description of care workers

Profession	Number of care workers	Years working at RCF	Number of care workers	Age	Number of care workers	Gender	Number of care workers
Social care worker: Activity leader	1	Less than 1 year	3	21–30 years	1	Female	9
Social care worker: Assistant nurse	2	1–3 years	3	31–40 years	2	Male	2
Social care worker: Activity leader and assistant nurse	1	4–6 years	1	41–50 years	5		
Healthcare worker: Nurse	3	More than 6 years	4	51–60 years	3		
Healthcare worker: Occupational therapist	2						
Healthcare worker: Physiotherapist	2						

All participants received both oral and written information about the study. The study was approved by the Swedish Ethical Review Authority (diary number: 2020–06643) prior to participant recruitment. Interested older adults and care workers informed their respective managers, who then suggested potential candidates based on the inclusion criteria. Final participant selection was confirmed by the research team.

### Data collection

Data were collected through semi-structured walking interviews [39] guided by open-ended questions based on the principal model of the four zones of contact with the outdoors [33]. The walking interview format enabled participants to reflect on their experiences in the environments where everyday activities and care and rehabilitation occurred. The interview guide explored participants’ experiences of each zone, the transitions between them, and how they navigated their use for activities and care and rehabilitation.

For both older adults and care workers, interview guides were developed to reflect the study’s dual focus on individual needs and professional practices. For older adults, the interview guide was pilot tested with two residents from an RCF outside the study sample, leading to minor linguistic refinements. The walking interviews with older adults were conducted during the summer of 2022 and included visits to zones 1–3, with additional discussions about Zone 4. Zone 4 was not physically visited due to participants’ fragile health and potential variations in travel time caused by distance. Efforts were made throughout the interviews to ensure participants felt comfortable and safe, with adjustments tailored to the individual situations. These walking interviews lasted between 28 and 80 min, were audio recorded, and transcribed verbatim.

The care workers’ interviews followed an interview guide focusing on their experiences with each zone and the transitions. The focus group format enabled care workers to discuss and compare their experiences and practices related to outdoor activities and care and rehabilitation. Conducted in spring 2022, these interviews also included visits to zones 1–3 and conversations about Zone 4. The care workers’ interviews lasted between 80 and 96 min, and were also audio recorded, and transcribed verbatim.

### Data analysis

The data were analysed specifically to address the present study’s research aim, with a focus on everyday activities, care, and rehabilitation in relation to Zones 1–4 of the zone model [33]. The analysis was conducted in several steps. First, the transcripts were read repeatedly to gain an overall understanding of the data. Second, data concerning everyday activities, care, and rehabilitation were identified deductively and organised according to Zones 1–4. Third, the identified activities and care and rehabilitation practices were classified according to whether they were described as currently taking place or as potential practices that participants considered possible, suitable, or desirable uses of the outdoor environments. Finally, data describing perceived benefits and barriers related to outdoor everyday activities, care, and rehabilitation were identified and organised separately as benefits and barriers.

## Results

The results describe how Zones 1–4 may support everyday activities, care, and rehabilitation, and the perceived benefits and barriers to their use in health service delivery. Findings are presented according to Zones 1–4 in the zone model.

### Zone 1: Indoor environments with windows

#### Everyday activities in zone 1

Older adults and care workers described a wide range of activities taking place in Zone 1, i.e. indoor areas with visual access to the outdoors via windows. These activities include both everyday routines and meaningful engagements that contribute to well-being and social interaction. Common activities in Zone 1 were attending church services, playing bingo, chatting with fellow residents, drinking coffee in the apartment, and eating meals in the dining area or restaurant. Many older adults spent time listening to music or the radio, watching TV, reading, or engaging in creative tasks such as painting with watercolours, solving crosswords, sorting items, folding napkins, or working on puzzles. Activities like singing, playing ball games, participating in music quizzes, and watching performances were also common in Zone 1. Time spent with relatives or friends was highly valued, whether through in-person visits or phone calls. Nature contact was facilitated indoors by gazing out of large windows to observe weather changes and seasonal shifts, or by sitting by an open balcony door to feel fresh air and hear sounds from outside. Care workers would sometimes open windows to allow older adults to enjoy outdoor music performances from indoors. Flowers and greenery were brought inside, either bought or picked, for decoration, and older adults also participated in planting seeds or repotting plants. Zone 1 provided opportunities for rest and relaxation, such as watching the view of trees, buildings, or the patio outside. Overall, these indoor environments were used flexibly to support both individual and shared activities that maintain social connection, stimulation, and a sense of connection to the outdoor environment.

#### Care and rehabilitation in zone 1

Care and rehabilitation activities were described as an integral part of daily routines performed in Zone 1. These routines included blood sampling, medication distribution, and wound care, typically performed by nurses. In addition, care workers reported administrative responsibilities, such as documentation, as part of their regular duties. Physical activities and rehabilitation activities in Zone 1 included exercises aimed at improving or maintaining strength, mobility, and balance. Common examples were walking and sit-to-stand training using a barre, corridor walking, breathing exercises, and hand and foot cycling using Manuped equipment. Group-based sessions and physical activity in shared Zone 1 spaces, such as the gym or training room, were also mentioned. Some adults participated strength exercises using fitness equipment, either independently or with support from care workers, typically physiotherapists. Assessment, prescription, and training with assistive devices were also common activities carried out by occupational therapists and physiotherapists.

Despite these opportunities, many older adults reported that few activities were offered in Zone 1. They described limited opportunities to engage in activities and uncertainty about how to occupy their time. This lack of activity contributed to feelings of boredom and under-stimulation.

### Zone 2: Entrances, balconies, patios, and conservatories

#### Everyday activities in entrances in zone 2

Entrance activities in Zone 2 i.e. the transitional area between indoors and outdoors, included practical activities like mail delivery and checking deliveries, as well as observing loading and unloading. Older adults also took on a “doorman” role by opening doors and socialized with relatives and acquaintances while sitting on benches, especially in summer. The entrance area was used to invite relatives and acquaintances for COVID-19 tests and served as a waiting spot for transportation services.

#### Everyday activities at balconies, patios, and conservatories in zone 2

Activities on the balconies, patios, and conservatories, also Zone 2 areas, were described as important parts of daily life, contributing to social interaction, relaxation, and engagement with nature. These activities included enjoying the sun and fresh air, appreciating natural elements such as wind and views, and observing surroundings including children playing, passersby, and garden life. Older adults engaged in various everyday activities such as chatting, socializing with family and friends, hosting celebrations, and meeting with relatives. Coffee breaks, eating meals, and barbecues were also common ways to spend time together in these spaces. Leisure and creative activities were reported, including reading, solving crosswords, painting or sculpting with clay, and working on crafts. Some older adults enjoyed meditating or reflecting while others took time to rest or sleep outdoors. Everyday activities included playing ball games (sitting in a circle and tossing a ball to one another), standing by the balcony railing, and gardening in planting boxes. Additional activities mentioned were smoking, listening to music entertainment, and offering bingo sessions.

#### Care and rehabilitation in zone 2

Care and rehabilitation activities in Zone 2 included medical history taking (anamnesis), follow-up meetings, and health conversations, which facilitated personalized care and ongoing assessment of older adults’ needs. Physical exercises aimed at maintaining or improving mobility and strength were common. These included balance and strength exercises, breathing exercises, hand training, standing exercises using balcony railings, group training sessions, gymnastics exercises, hand and foot cycling using Manuped equipment, and outdoor walking training. Functional walking assessments and inspection of mobility aids were also suited activities for Zone 2. Sitting outdoor together, older adult and care worker, could serve as a calming activity to help reduce anxiety. Clinical tasks, such as blood sampling, could also be performed within this zone.

Despite these opportunities, both older adults and care workers described limited use of Zone 2. It was often characterized as a passive space, with few organized activities. Older adults primarily remained seated, and social interactions, such as coffee breaks, were infrequent, contributing to feelings of under-stimulation and a lack of meaningful engagement.

### Zone 3: Gardens

#### Everyday activities in zone 3

Activities in Zone 3, i.e. gardens, were described as vital parts of daily life, contributing to relaxation, social interaction in the garden café, and connection with nature. These activities included enjoying the sun and fresh air, appreciating greenery, blooming flowers, and natural elements like wind and views. Older adults observed animals, including birds, deer, horses, cows, fish, and toads, as well as other persons and the surroundings. Social engagement was central, with older adults attending celebrations such as Midsummer, socializing with relatives and acquaintances, and participating in spontaneous conversations. Shared moments included drinking coffee, hot chocolate, and enjoying buns outdoors, even during snowy weather, and eating meals together. Barbecues also featured as popular ways to spend time collectively. Leisure activities varied widely, including reading, listening to music or the radio, and enjoying music entertainment or performances on outdoor stages. Games and gentle exercise were common, from playing ball, boules, and bowling, to walker races (a walking competition using mobility aids), walking, and riding around in wheelchairs while observing the surroundings. Sitting in garden swings or resting outdoors provided moments of calm and comfort. Gardening with care workers, planting, and tending to plants encouraged meaningful engagement with the environment, sometimes including cognitive stimulation like trying to remember plant names. Taking walks around the building or in the garden further supported physical activity and observation of the natural surroundings.

#### Care and rehabilitation in zone 3

Medical tasks such as anamnesis, health conversations, and outdoor follow-up meetings or evaluations were conducted in Zone 3, often integrated into walks or sitting interactions. Blood sampling could also be performed outdoors. Physical and rehabilitation activities focused on maintaining or improving mobility, balance, and endurance. These included walking training, balance exercises (e.g., reaching for natural elements), standing exercises using railings, and hand and foot cycling using Manuped equipment. The gardens could be used for functional training, such as walking on slopes, sit-to-stand exercises at benches, and stair training. Resting spots, like benches placed along walking paths, supported pacing and recovery during training sessions. Older adults also engaged in training, such as wheelchair driving or cycling around the building. More structured activities included functional walking assessments and inspection of mobility aids.

Despite these opportunities, both older adults and care workers described limited use of Zone 3. Organized everyday activities and care and rehabilitation were rare, particularly during the autumn and winter months.

### Zone 4: Surroundings

#### Everyday activities in zone 4

Everyday activities in Zone 4, i.e. the surroundings beyond the RCFs, enabled older adults to maintain everyday life, social contact, and engagement with the surrounding society. Outings often included walks, bike rides in pairs (duo bikes), or motion and exercise, with possibilities to rest on a walker or observe the surroundings along the way. Use of transportation services, such as car trips, bus or tram rides, made it possible to visit shops, kiosks, restaurants, cafés, and the harbour, often accompanied by relatives or care workers. Shopping, going out for coffee, or sitting in the square to talk to people provided informal opportunities to socialize and interact with acquaintances. Other meaningful activities included visiting relatives or acquaintances, traveling to natural areas, visiting doctors, attending funerals, placing bets on horses, and smoking.

#### Care and rehabilitation in zone 4

Zone 4 offered both implemented and potential opportunities for care and rehabilitation activities in the surroundings. Medical tasks, such as anamnesis and health conversations or evaluations, were sometimes conducted outdoors during walks. Physical and rehabilitation activities in Zone 4 included duo cycling and wheelchair driving, as well as functional walking training. These activities supported mobility, and engagement with the broader surroundings. Several reflections emphasized that the surroundings could be a good place for care and rehabilitation activities, depending on older adults’ needs and care workers involvement.

Despite these opportunities, older adults described that Zone 4 was sometimes perceived as unstimulating. Repetitive walking routes contributed to feelings of boredom, and many older adults expressed a need for more diverse and engaging outings beyond the RCFs grounds.

Table 3 presents the identified everyday activities and care and rehabilitation practices, organised by zone. The results are further detailed by distinguishing between participant groups and between practices that occurred and those identified as potential practices.

**Table 3:** Everyday activities and care and rehabilitation in zones 1–4

Zone	Everyday Activities	Care and Rehabilitation
Zone 1	- Attend church service^1^ - Bingo^1^ - Buy or pick flowers^2^ - Chat with acquaintances and tenants^1^ - Check the weather and observe nature through windows^2^ - Drink coffee^1,2^ - Eat meals^1^ - Fold napkins^1^ - Gaze out the window^1^ - Listen to music (individual/entertainment)^1^ - Listen to outdoor music through open windows^2^ - Listen to the radio^1^ - Look at TV^1^ - Open windows for fresh air/sounds^2^ - Paint with watercolours^1^ - Phone calls^1^ - Plant seeds/repot plants^2^ - Play ball games^1^ - Puzzle^1^ - Read^1^ - Rest^1^ - Sing^1^ - Sit by the open balcony door and look out^2^ - Socialize with relatives/friends^1^ - Sort items in boxes^1^ - Solve crosswords^1^ - Take part in a music quiz^1^ - Talk^1^ - Watch performances^1^ - Watch the view (trees/buildings)^1, 2^ - Watch what is happening outside (patio)^2^	- Assessments and prescription^2^ - Balance, walking, and sit-to-stand training using a barre^2^ - Blood tests^2^ - Breathing exercises^1^ - Documentation and administration^2^ - Group exercise^1^ - Hand and foot cycling^2^ - Medication distribution^2^ - Physical activity^1^ - Strength training with equipment (training room)^2^ - Training in the apartment^1^* - Training in the corridor^1^ - Training in the gym^1, 2^ - Wound care^2^
Zone 2	*Entrance*: - Act as a courier^1^ - Check deliveries^1^ - Invite people to COVID-19 test^1^ - Observe loading/unloading^1^ - Open entrance door for others^1^ - Sit on bench^1^ - Socialize with relatives/acquaintances^2^ - Wait for transportation^2^ *Balcony*,* Patio*,* Conservatory*: - Appreciate sun, wind, presence of children^1, 2^ - Barbecue/Grill evenings^1, 2^ - Be alone on the patio^1^ - Bingo^2^* - Chat/Talk^1^ - Coffee break/Drink coffee or snack^1, 2^ - Eat meals^1, 2^ - Enjoy time together^1^ - Enjoy the sun/Sunbathe^1, 2^ - Experience nature and view^1^ - Family visits^1^ - Gardening in planting boxes^2^ - Gaze at sun, clouds, surroundings^1^ - Host celebrations^2^ - Listen to music entertainment^2^ - Meet with relatives^1, 2^ - Meditate or reflect^1^ - Observe surroundings^1^ - Paint or sculpt with clay^1^ - Plant, care for, or water flowers^1, 2^ - Read^1^ - Rest^1^ /Sleep in outdoor bed^1^* - Sit at table and sunbathe^1^ - Socialize/Spend time with others^1^ - Smoke^1, 2^ - Solve crosswords^1^ - Stand by balcony railing^1^ - Throw/play ball games^1^ - Watch car license plates^1^ - Watch children playing, passersby, dogs, greenery, street^1^ - Work on crafts^1^	- Anamnesis (medical history taking) ^2^* - Balance and strength exercises^2^ - Breathing exercises^1^ - Follow-up meetings^2^ - Functional walking assessments^2^* - Group training (standing exercises using balcony railing)^1^*^, 2^ - Gymnastics exercises^2^* - Hand and foot cycling^2^* - Hand training^2^* - Health conversations^2^* - Inspection of mobility aids^2^* - Outdoor walking training^2^* - Sitting together to reduce anxiety^2^ - Taking blood/other samples^2^* - Training^1, 2^*
Zone 3	- Attend celebrations^1^ - Barbecue^1, 2^ - Breathe fresh air^1^ - Boules^1, 2^ - Bowling^2^ - Celebrate Midsummer^1, 2^ - Drink coffee, hot chocolate, buns outdoors (snowy weather)^2^ - Eat meals^1, 2^ - Enjoy greenery, nature, fresh air^1^ - Exercise/move around^1^ - Gardening with care workers^2^ - Go for a walk^1, 2^ - Go up and down a hill^1^ - Listen to music^1^ - Listen to the radio^1^ - Look at animals^1, 2^ - Look at birds^1^ - Look at blooming flowers^1^ - Look at fish in the pond^1^ - Look at people^1^ - Look at plants, remember names^1^ - Look at fish and toad in the pond^2^ - Look at surrounding forest^2^ - Look at wild animals^1^ - Music entertainment^2^ - Music on outdoor stage^2^ - Observe surroundings/view^1^ - Outdoor café^1, 2^ - Plant^2^ - Play games^1^ - Read^1^ - Rest^1^ - Ride around in wheelchair and observe surroundings^1^ - Rollator race^2^ - Sit in garden swing^1^ - Socialize with acquaintances^1^ - Socialize with family or friends^1^ - Spend time with others^1^ - Spontaneous conversation/socializing^1^ - Sunbathe^1, 2^ - Take a walk around building^1^ - Throw a ball^2^ - Walk in garden and observe^1^ - Watch entertainment (orchestras, troubadours)^1, 2^	- Anamnes (medical history taking)^2^* - Balance training (reaching, smelling, feeling nature)^2^ - Cycling around the building^2^ - Functional walking assessments^2^* - Hand and foot cycling^2^* - Inspection of mobility aids^2^* - Health conversations, evaluations, follow-ups^2^* - Physical training^2^ - Resting (benches along paths)^2^ - Stair training^2^ - Standing exercises using railings^2^ - Taking samples^2^* - Training^1^*^, 2^ - Walking training (walking slopes*, sit-to-stand at benches)^2^ - Wheelchair driving^1, 2^
Zone 4	- Attend a funeral^1^ - Attend a senior exercise class^1^ - Car trip^1^ - Explore the surroundings^1^ - Go for a walk^1, 2^ - Go out for coffee^1^ - Go shopping (with/without relatives/care workers)^1, 2^ - Interact with acquaintances at store or park^1^ - Motion/exercise^1^ - Observe surroundings^1^ - Place bets on horses^1^ - Rest while sitting on a walker^1^ - Ride a duo bike^1^ - Ride in car, bus, or transportation^1^ - Sit in square and talk to people^1^ - Smoke^2^ - Socialize with family or acquaintances^1^ - Take a tram ride^2^ - Travel to natural area^1^ - Visit café with relatives^2^ - Visit kiosk^1^ - Visit relatives or acquaintances^1^ - Visit doctor^1, 2^ - Visit shops and squares^1, 2^ - Visit harbour^1^ - Visit restaurant^1^	- Anamnes (medical history taking)^2^* - Duo cycling^2^ - Evaluations^2^* - Functional walking training^2^* - Health conversations^2^** - Training^1^*^, 2^ - Wheelchair driving^1, 2^

^1^ = reported by older adults; ^2^ = reported by care workers. * = activities or care and rehabilitation practices identified by older adults or care workers as potential practices, rather than practices currently taking place

### Benefits and barriers to outdoor everyday activities and care and rehabilitation

The older adults experience that engaging in everyday activities is both meaningful and enjoyable, providing a sense of purpose and community. Care workers emphasize the advantages of being outdoors, as these environments offer fresh air and close contact with nature, experiences that cannot be fully replicated indoors. Both the older adults and the care workers perceive the outdoor environments as more stimulating than indoor environments. While both indoor and outdoor environments are important for meaningful activities, outdoor activities have the potential to improve joy and overall well-being according to the older adults and care workers.

Outdoor everyday activities and care and rehabilitation in RCFs provide a welcomed change of environment highly valued by both the older adults and care workers. Spending time outdoors encourages more frequent engagement, increases energy and alertness, and promotes positive thinking, benefiting both body and mind. Fresh air, physical exercise, recreation, and sensory stimulation are central aspects of outdoor activities or interventions.

Care workers reported that they also experienced their work environment as more stimulating and enjoyable when activities or care and rehabilitation take place outdoors compared to cramped, windowless indoor spaces. One care worker highlighted that a day without outdoor exposure feels incomplete, underscoring the fundamental role of outdoor environments in supporting health and well-being.

At the same time, everyday activities and care and rehabilitation in Zones 2–4 for older adults face several barriers that limit participation and reduce potential health benefits. Physical constraints such as insufficient and poorly maintained outdoor environments, uneven surfaces, narrow doors, heavy doors without automatic openers, and inadequate elevator facilities hinder accessibility, especially for those using mobility aids. Seasonal challenges, including difficulty dressing appropriately and lack of suitable outdoor footwear, further restrict outdoor engagement.

Informational and organizational barriers also play a significant role. Older adults often lack orientation to outdoor environments and clear information about available everyday activities, while care workers face time constraints and uncertainty about how they can use outdoor environments for activities, care, and rehabilitation. Gaps in care workers’ knowledge of mobility assistance to older adults, combined with limited personalized support, further restrict opportunities for safe and comfortable outdoor participation.

Social aspects also influenced engagement, as some older adults expressed a preference for being outdoors with others, describing being outdoors alone as less enjoyable and associated with feelings of loneliness. Additionally, environmental issues such as traffic noise, vandalism, and absence of amenities (e.g., seating, warming facilities) diminish the attractiveness of everyday activities and care and rehabilitation outdoors.

## Discussion

Drawing on the same original empirical data as two previous publications [42, 43], this secondary analysis contributes a complementary perspective by examining how different zones of contact with outdoor environments can support everyday activities and person-centred care and rehabilitation for older adults in RCFs. While the previous publications addressed other aspects of the participants’ experiences, the present analysis focuses specifically on the use of outdoor environments in everyday activities, care, and rehabilitation, as well as perceived benefits and barriers to their integration into health service delivery.

Zones 1–4 offered different, partly overlapping opportunities. Oder adults and care workers described social interaction, meals, physical activity, and contact with nature, while care workers additionally identified opportunities to integrates outdoor environments into care and rehabilitation. These opportunities can be adapted to older adults’ personal needs, preferences, functional abilities, and goals, which is central to a person-centred approach [30–32]. However, available opportunities did not necessarily translate into participation or routine use. Participants described limited organised activities and several barriers, including physical, seasonal, organisational, informational, and social aspects. Thus, access to outdoor environments alone is insufficient. Their use requires access to accessible environments, adequate organisational conditions, and care workers with the time, knowledge, and support needed to facilitate participation.

Outdoor environments may also provide a more stimulating and enjoyable physical work environment for care workers. At the same time, uncertainty about how to integrate outdoor environments into care and rehabilitation, together with organisational constraints, may limit their use. The lack of organised outdoor activities, care, and rehabilitation therefore points to a need to include outdoor engagement into RCF routines rather than treating it as an optional or ad hoc component. This is consistent with national data showing that 92% of Swedish RCFs lack established routines for regular outdoor activities, care, and rehabilitation [36].

The different zones may support different activities, care, and rehabilitation depending on older adults’ needs and purpose of the activity. Zone 1 enabled activities, care, and rehabilitation indoors while providing visual contact with the outdoors. Zones 2 and 3 provided opportunities for outdoor engagement close to the RCFs, while Zone 4 enabled participation beyond the RCF grounds. Table 3 provides a consolidated overview of the identified activities and care and rehabilitation practices and may serve as a practical source of inspiration for care workers and managers in RCFs when considering how outdoor environments can be used. Such use should be guided by the individual older adult’s needs and wishes rather than by the availability of a particular zone.

Overall, outdoor environments can support everyday activities and person-centred care and rehabilitation in RCFs, but their potential is not automatically realised. The main contribution of this study is to show both the range of opportunities offered across the four zones and the aspects that influence their use. Integrating outdoor environments into routine health service delivery therefore requires not only physical access but also organisational support and care practices that enable their use to respond to older adults’ needs and wishes. This has implications for RCF design, care worker training, organisational routines, and policy development.

### Methodological considerations

A strength of the methodological approach was the use of complementary data collection methods and the zone model, which together enabled exploration of participants’ experiences from both individual and professional perspectives and in relation to different environmental contexts. Walking interviews [39] allowed participants to reflect on their experiences while moving through or observing the environments under study, thereby linking their accounts to specific environmental features. This was particularly relevant given the older adults’ varying mobility and functional capacity. The focus group format [40] used with care workers enabled participants to discuss professional experiences and practices, providing insight into how outdoor environments may be integrated into everyday activities, care, and rehabilitation. The zone model [33, 35] provided a structured framework for organising the data according to different types of contact with the outdoors. This facilitated examination of everyday activities, care, rehabilitation, and perceived benefits and barriers in relation to each zone.

Several limitations should be considered. Purposive sampling may have introduced selection bias, as participants who were more engaged in outdoor activities or more comfortable participating in interviews may have been overrepresented [38]. The inclusion of only three RCFs limits the range of environmental characteristics represented. Finally, although walking interviews enabled reflection in the environmental contexts under study, Zone 4 was not physically visited because of older adults’ physical limitations and potentially long travel distances. This may have limited the depth of data concerning the most distant outdoor environments. Despite these limitations, the study provides insights into the benefits and barriers associated with using outdoor environments in RCFs. Although conducted in a Swedish context, the findings may also be relevant to RCFs internationally, particularly regarding organisational, environmental, and workforce-related conditions for outdoor engagement in long-term care settings [21, 44].

Future research should investigate how the identified benefits and barriers can be addressed in practice and how outdoor environments can be systematically integrated into everyday activities, care, and rehabilitation. Studies across a larger and more diverse range of RCFs, including intervention and implementation studies, are needed to examine how such integration can be established and sustained.

## Conclusion

Outdoor environments represent a valuable but underutilized resource in RCFs. The findings demonstrate that all four zones can support a broad range of everyday activities and person-centred care and rehabilitation, with opportunities that can be adapted to older adults’ needs and wishes. However, these opportunities are not automatically realised, as environmental, organisational, informational, seasonal, and social barriers constrain their use. Integrating outdoor environments into routine health service delivery therefore requires access to environments, supportive organisational routines, and care workers with the time, knowledge, and support needed to facilitate their use.

## Data Availability

Not applicable.

## Declarations

### Generative AI in the manuscript preparation process

During the preparation of this work the authors used ChatGPT in order to improve texts. After using this service, the authors reviewed and edited the content as needed and take full responsibility for the content of the published article.

## Abbreviations

RCF: Residential care facility
